# The effect of cobalt alloying on the phase transformation kinetics of Ni-Ti alloys

**DOI:** 10.1016/j.heliyon.2024.e37390

**Published:** 2024-09-03

**Authors:** Rocco Puopolo, Sally Ruschendorf, Ajai S.K. Thadayil, Scott Cook, Mert Celikin

**Affiliations:** a Materials Design and Processing Laboratory, School of Mechanical and Materials Engineering, University College Dublin, Ireland; b Boston Scientific, Research and Development, Galway, Ireland; c SFI I-Form Advanced Manufacturing Research Centre, Ireland

**Keywords:** Ni-Ti alloys, Cobalt alloying, Shape memory alloys, Phase transformation, X-ray diffraction, Differential scanning calorimetry

## Abstract

This study investigates the influence of cobalt (*Co*) alloying addition and heat treatment temperature on the phase transformation behaviour controlling the superelasticity and shape memory effect (SME) of Nickel-Titanium (Ni-Ti) alloys, commonly known as nitinol. The microstructural evolution upon heat treatment conducted at a temperature ranging from 440 to 560 °C was thoroughly analyzed via Differential Scanning Calorimetry (DSC), X-ray Diffraction (XRD), and Scanning Electron Microscopy/Energy Dispersive Spectroscopy (SEM/EDS). Increase in heat treatment temperatures from 470 °C up to 530 °C led to the dissolution of particles present in as-received (cold-worked) condition. It was determined that *Co* addition into the Ni-Ti alloy system resulted in a change in the nucleation and growth kinetics of Ti-rich precipitates, leading to the formation of larger and fewer particles during processing. Both binary and ternary alloys showed a decrease in austenite finish temperature (A_f_) with increasing heat treatment temperatures, however, the rate of decrease was found to be higher for *Co* containing ternary alloys. This is linked with faster structural relaxation when Co is present and evidenced by lattice size variation during heat treatment. It is highlighted that heat treatment methodology needs to be tailored to the specific alloy composition for controlling superelasticity and SME via alloy design.

## 1 Introduction

Binary nickel-titanium alloys (with 48–52 at% nickel), commonly referred to as nitinol, are renowned for their unique properties such as the shape memory effect (SME) and pseudoelasticity, rendering them indispensable in biomedical and aerospace applications [1,2]. The SME, characterized by the ability to revert heavily deformed shapes to their original form upon heating, enables nitinol's use as actuators [1]. Additionally, nitinol's pseudoelasticity allows it to elastically deform up to 8 % strain due to stress-induced austenite to martensite phase transformation, compared to most metals which are limited to around 0.5 % elastic strain recovery [3]. However, for various critical applications such as biomedical implants, aerospace components, and industrial actuators, improved mechanical properties along with tailored phase transformation temperatures are required.

A reversible transformation between the austenite-*A* (cubic-B2) parent phase and martensite-*M* allows pseudoelasticity and the shape memory effect (SME) [[4], [5], [6], [7]]. Under certain conditions, the formation of a third phase, known as the *R*-phase, is also possible. The R-phase is characterized by a rhombohedral distortion of austenite [4]. Similar to the martensitic phase, it has different variants that can accommodate each other [8]. R-phase formation requires less energy compared to martensitic transformation, leading to competition for the succession of austenite. While martensite is usually thermodynamically favoured, the R-phase can form if it is kinetically preferred [4]. Coherent precipitates and dislocation networks suppress the martensitic transformation, with R-phase transformation occurring after thermomechanical treatments. However, the coarsening of precipitates, leading to the loss of coherency, will hinder the R-phase transformation [9].

The production of nitinol parts is complex, and each processing step, including hot and cold working coupled with heat treatment, can significantly impact the final material properties [[10], [11], [12], [13]]. The alloy transformation temperatures are primarily determined by the Ni/Ti ratio in the alloy. For instance, a 1 % change in the amount of either Ni or Ti can cause a 100 °C shift in the alloy transition temperatures [11]. The resulting parts are then heat-treated at temperatures between 300 °C and 550 °C to optimize the transformation temperatures [12,14]. The formation of precipitates such as Ni₃Ti, or Ti₂Ni during processing and/or heat treatment affects the matrix composition and influences transformation temperatures. Furthermore, the type and orientation of specific precipitates promote the growth of specific variants of martensite and R-phase [3,6]. The transformation temperatures of nitinol are highly sensitive to changes in internal stress as well as the Ni/Ti ratio of the matrix; for example, the *Af* and *Mf* temperatures increase when Ni concentration decreases [10,11,15].

Numerous alloy development studies have been conducted to improve mechanical performance and control phase transformation temperatures, including the austenite finish temperature (Af), which should vary depending on the specific application. For example, to optimize the functionality of nitinol for biomedical stents that operate isothermally at 37 °C and allow for self-expansion, the Af temperature must be lower than human body temperature. Therefore, when the device is deployed in the artery, it remains in the austenitic, superelastic form [15,16]. Ternary elements such as iron (*Fe*), chromium (*Cr*), copper (*Cu*), zirconium (*Zr*), hafnium (*Hf*), vanadium (*V*), and cobalt (*Co*) have been used to modulate nitinol behaviour [5,17,18]. *Fe* or *Cr* can be added to reduce the transformation temperatures of Ni-Ti alloys. *Cu* is commonly used to reduce and stabilize pseudoelastic hysteresis [17]. When 10 at.% *Cu* is added, phase transformation via the *R*-phase can be achieved. Additionally, the martensite start temperature (Ms) and hysteresis become more resistant to changes in the Ni/Ti ratio, and the formation of Ni₄Ti₃ precipitates is suppressed with *Cu* addition. On the other hand, *Zr* and *Hf* alloying increase the solubility, resulting in the formation of coherent precipitates and a decrease in *Ms.* Similarly, *V* addition leads to solid solution strengthening, which improves pseudoelastic behaviour and the SME. However, *V* can also form secondary phases with either *Ni* or *Ti* that can oxidize and deteriorate the SME [18].

In this study, we focus on the effect of cobalt (*Co*) alloying, which is known to enhance mechanical performance compared to traditional nitinol. For example, Ni-Ti-Co (*NiTiCo*) alloys exhibit a higher modulus, an increased loading and unloading plateau by 30 % [19], and a decreased martensite start temperature (*Ms*) [5,20]. Significant properties such as biocompatibility, fatigue strength, and corrosion resistance are comparable to those of the binary alloy [19,21]. Similar to iron (*Fe*) and chromium (*Cr*) alloying, *Co* can suppress the formation of martensite, resulting in the formation of the R-phase [4].

The material transformation temperatures are influenced by the addition of *Co*, depending on the specific material composition, atomic configuration, and precipitates. Hosoda et al. [5] reported that an increase in *Co* content decreases both the austenite start temperature (As) and Ms. An increase of 1 mol% *Co* leads to a decrease of both *As* and *Ms* by 15–20 °C. Jing et al. [22] heat-treated the samples at 500 °C for 1 h after hot working, and their results showed that the transformation temperatures decreased with increasing *Co* addition. A study conducted by Fasching et al. [19] demonstrated the critical importance of thermomechanical treatment for controlling transformation temperatures. By adding *Co*, the austenite transformation temperature in the ingot varied by almost 60 °C. However, after manufacturing and final processing with optimized heat treatments, the *Af* temperature of both *NiTiCo* (11 °C) and binary *NiTi* (7 °C) were similar. Although the role of *Co* addition in mechanical performance is well established, the effect of *Co* alloying on microstructural evolution upon heat treatment and the corresponding changes in phase transformation temperatures remains ambiguous.

This study aims to understand the relationship between microstructural evolution and the phase transformation kinetics of *NiTiCo* alloy in comparison to nitinol, both in as-received (cold-worked) and heat-treated conditions at temperatures ranging from 440 to 560 °C. Understanding the link between microstructural evolution and transformation temperatures will provide insights into controlling superelasticity and the shape memory effect (SME) through alloy design.

## 2 Experimental Procedure

The *NiTi* and *NiTiCo* alloys studied were provided by Boston Scientific in a cold-worked condition. The samples were hollow tubes with an outer diameter of 10 mm and a wall thickness of 0.6 mm. To investigate the effect of different heat treatment conditions, the as-received samples were heat treated at temperatures ranging from 440 °C to 560 °C for 1 h. This temperature range was chosen based on available literature [2,11], but we expanded it to gain a clearer understanding of the evolution of the austenite finish (*Af*) temperature, microstructural changes, and the effect of *Co* alloying (Table 1).

**Table 1:** Summary of specimens and conditions.

Specimen name	Heat Treatment Temperature [°C]
*NiTi AR*	As-received
*NiTi 440*	440
*NiTi 470*	470
*NiTi 500*	500
*NiTi 530*	530
*NiTi 560*	560
*NiTiCo AR*	As-received
*NiTiCo 440*	440
*NiTiCo 470*	470
*NiTiCo 500*	500
*NiTiCo 530*	530
*NiTiCo 560*	560

Heat treatment was performed using a Nabertherm B400/410 box furnace (±2 °C) with a heating rate of 5 °C/min to ensure thermal equilibrium between the sample and the furnace during the heating cycle. The samples were water quenched upon heat treatment, and a titanium oxide/dioxide layer formed on the surface of the specimens, giving them a unique color depending on the heat treatment temperature and composition.

The hollow tubes were cut using a Buehler IsoMet High-Speed Pro machine. For microstructural and mechanical analysis, as well as X-ray diffraction (XRD) measurements, 15 mm long tubes were cut using a diamond blade at a rotation speed of 400 rpm. The circular cross-sectional surface of the samples was prepared using metallography techniques. To prepare samples for XRD measurements, the long side of the tubes was ground to create a plane surface area. The specimens were secured using a vice, and a high-carbon steel rasp was used to create a flat surface along the axis of the cylinder.

X-ray diffraction (XRD) analysis was conducted using a Rigaku SmartLab X-ray diffractometer equipped with Cu-Kα radiation (λ = 1.5406 Å). The experiment was performed in one-dimensional (1D) scan mode, covering the 2θ range from 30° to 90° with a step size of 0.01° and a scan speed of 20° per minute. Samples were mounted on an ASC 6 attachment, and data acquisition was performed using Rigaku's SmartLab Studio II software. The Lorentzian function was used for peak fitting to identify the exact peak position and the full width at half maximum (FWHM).

ImageJ software was used to determine the average particle size and the average area occupied in the matrix of the *NiTi* and *NiTiCo* samples heat treated at 470 °C, 500 °C, and 530 °C [23]. SEM BSE images from multiple regions were used to measure the average particle size, based on 250–400 particles per condition. AztecOne software was used for EDS data analysis, where around 10 particles per sample were identified in random locations of the specimen and analyzed. For each sample, 2 different locations were chosen to analyze the composition of the matrix.

Differential scanning calorimetry (DSC) measurements were conducted using a NETZSCH DSC 214 Polyma according to ASTM F2004-17 to determine the phase transformation temperatures before and after heat treatment. According to the standard, a sample size between 25 and 45 mg was used. The temperature program started with heating to 90 °C with a ramp rate of 10 °C/min (isothermal holding for 5 min). Subsequently, the sample was cooled to −140 °C using a ramp rate of −10 °C/min (isothermal holding for 5 min) and then heated again to 90 °C at a rate of 10 °C/min.

## 3 Results

### 3.1 Particle size analysis by SEM

**Fig. 1:**
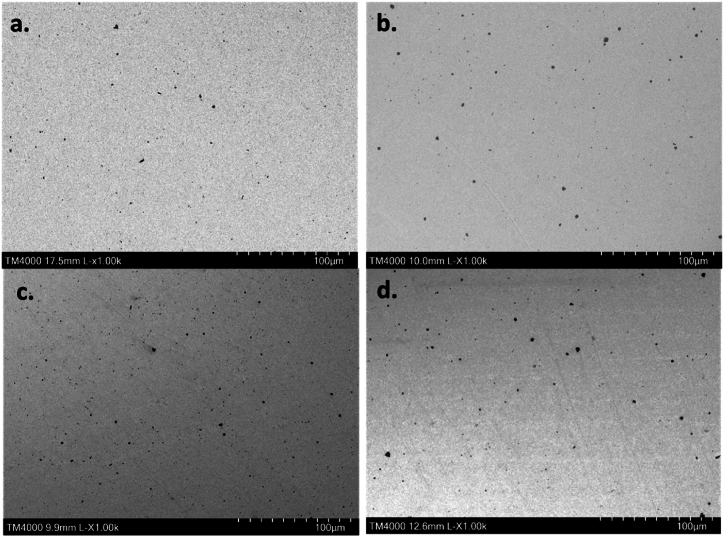
SEM- BSE micrographs of the as-received NiTi (a), NiTiCo (b) and NiTi 470 (c), NiTiCo 470 (d).

SEM analysis was conducted to quantitively evaluate the effect of *Co* addition and heat treatment temperature on the average particle size. A visual examination of the SEM micrographs of the as-received and 470 °C heat treated specimens (Fig. 1) shows the difference between binary (*NiTi*) and ternary alloys (*NiTiCo*), revealing that *NiTiCo* samples are characterized by the presence of larger and fewer particles. The average particle size and occupied area within the matrix are summarized in Table 2.

**Table 2:** Average particle size and particle proportion within the matrix.

Material	Particle Size [μm]	Particle Area Proportion in the Matrix [%]
*NiTi AR*	0.55	0.261
*NiTi 470*	0.68	0.287
*NiTi 500*	0.57	0.256
*NiTi 530*	0.30	0.245
*NiTiCo AR*	0.84	0.226
*NiTiCo 470*	1.1	0.249
*NiTiCo 500*	0.93	0.223
*NiTiCo 530*	0.77	0.212

**Fig. 2:**
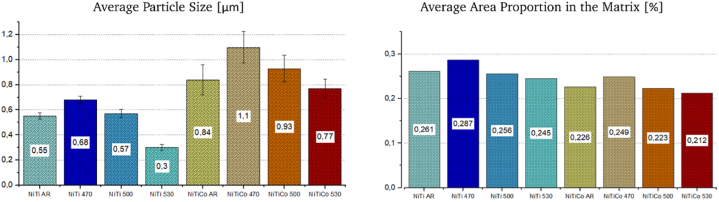
Average particle size and particle proportion within the matrix of the heat-treated samples.

Compared to the as-received condition, for both alloys (*NiTi and NiTiCo*) the particle size and the area occupied in the matrix increase when heat treated at 470 °C indicating further precipitation and growth of the existing particles (Table 2). However, at temperatures 500 °C and above, there were smaller particles occupying a lower percentage of the area in the matrix. The results suggest that the precipitates started to dissolve at higher heat treatment temperatures than 470 °C. The effect of the heat treatment temperature on average particle size and area proportion in the matrix is given in Fig. 2.

The comparison between *NiTi* and *NiTiCo* alloys reveals that the addition of *Co* significantly increases the size of the precipitates both in as-received and heat-treated conditions. However, the occupied area within the matrix decreases due to a reduction in the number of particles. Therefore, it can be stated that *Co* alloying addition led to a change in nucleation and growth kinetics of precipitate formation. A lower nucleation rate is present in as-received/cold-worked *NiTiCo* alloys in comparison to nitinol which caused fewer and coarser particles to form during hot working. Moreover, this effect might also be correlated with difference in grain size where grain boundaries act as nucleation sites. This is part of an on-going work focusing on grain size evolution during the hot working process and its effect on dynamic precipitation.

### 3.2 Particle and matrix compositional analysis by SEM/EDS

**Fig. 3:**
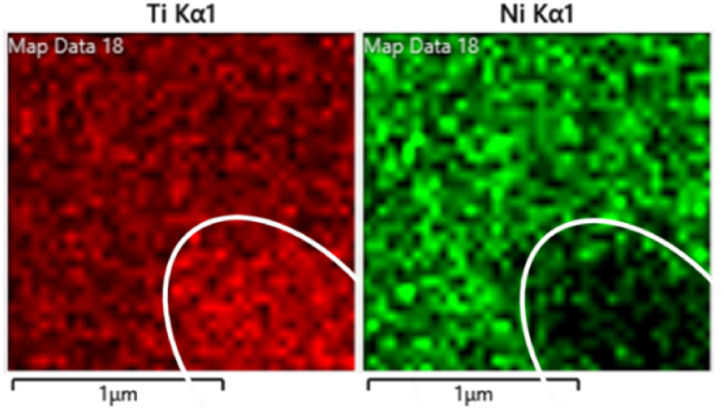
EDS-mapping of matrix and a representative Ti-rich precipitate in NiTiCo AR.

To analyze and compare the composition of the matrix and precipitates, SEM/EDS analysis was conducted (Table 3). The results can be used to evaluate the *Ni/Ti* ratio which governs the phase transformation temperatures of the system. The analysis of the matrix was based on sections without particles to determine the proportion of *Ni*, *Ti*, and *Co*. Similarly, 10 precipitates per sample were analyzed. To limit the matrix effect, EDS analysis was limited to the central region of the precipitates. According to the examination, only Ti-rich particles were determined (Fig. 3).

**Table 3:** Average composition of investigated matrices and particles.

Material	Structure	at%Ni	at%Ti	at%Co	*Ni/Ti ratio*
*NiTi AR*	Matrix	*51.6*	*48.4*	*-*	1.07
	Particles	*15.6*	*84.1*	*-*	0.18
	σ	*0.4*	*0.2*	*-*	–
*NiTi 470*	Matrix	51.8	47.8	–	1.09
	Particles	15.7	83.4	–	0.19
	σ	0.1	0.1	–	–
*NiTi 500*	Matrix	52.2	47.8	–	1.1
	Particles	15.7	83.6	–	0.19
	σ	0.2	0.4	–	–
*NiTi 530*	Matrix	51.8	48.2	–	1.07
	Particles	14.5	84.9	–	0.17
	σ	0.1	0.1	–	–
*NiTiCo AR*	Matrix	*50.4*	*47.7*	*1.4*	1.06
	Particles	14.4	85.4	–	0.17
	σ	0.1	0.4	–	–
*NiTiCo 470*	Matrix	50.8	47.5	1.3	1.07
	Particles	14.8	85	–	0.17
	σ	0.2	0.1	–	–
*NiTiCo 500*	Matrix	50.6	47.5	1.3	1.07
	Particles	15.4	83.8	–	0.18
	σ	0.1	0.3	–	–
*NiTiCo 530*	Matrix	50.1	48.3	1.1	1.04
	Particles	17.3	81.9	–	0.21
	σ	0.1	0.1	–	–

In both binary and ternary alloys, Ti content of the matrix increases with increasing heat treatment temperature. This is consistent with the SEM observations indicating the dissolution of Ti-rich particles upon heat treatment, probably due to a decreased stability of the precipitates at higher temperatures. Fewer particles were observed with increasing heat treatment temperature, suggesting that precipitates dissolved back into the matrix. On the other hand, *Co* content in the matrix was determined to be around 1.4 at% in the as-received condition, 1.3 at% when heat treated at 470 °C and 500 °C, and 1.1 at% when the final heat treatment was performed at 530 °C. The decrease could indicate more *Co* segregation onto precipitates upon high temperature heat treatment. However, no *Co*-containing precipitates were determined. This might be due to EDS resolution issues, where further investigation is necessary.

Ti-rich phases were present in all conditions and accounted for most of the observed precipitates. It can be seen from the examined sections (Fig. 1) that all observed particles are dark grey and therefore Ti-rich particles (Fig. 3) are probably Ti_2_Ni, similar to the previously observed particles in literature [24,25]. However, Ti-rich precipitates observed in this study have a Ni content varying between 14.5 at% and 17.3 at%. According to binary Ni-Ti phase diagram, the composition of the identified particles falls in a region where β-Ti and ${Ti}_{2}Ni$ coexist. This could suggest that the Ti/Ni atomic ratio varies throughout the precipitates and that the final composition is given by the average between the composition of the β-Ti and ${Ti}_{2}Ni$ phases present. Nevertheless, EDS is a semi-quantitative technique and further in-depth study via Transmission Electron Microscopy (TEM) is needed for detailed precipitate characterisation.

### 3.3 Differential Scanning Calorimetry (DSC)

**Fig. 4:**
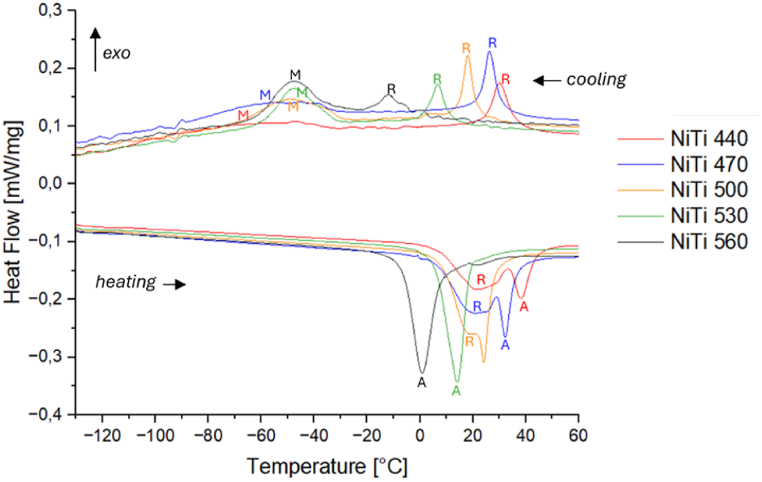
DSC curves of NiTi samples heat treated at 470 °C, 500 °C and 530 °C.

Upon cooling, the *M*_*s*_ and *M*_*f*_ and upon heating *A*_*s*_ and *A*_*f*_ can be determined. The transformation temperature peaks, *Mp* and *Ap*, represent the temperatures at which 50 % of the material transforms into martensite or austenite, respectively. Additionally, the *R-phase* start and finish temperatures can be determined using heating and cooling curves (upon cooling: *R*_*sc*_, *R*_*fc*_; upon heating: *R*_*sh*_, *R*_*fh*_). The temperature hysteresis of the material is defined as the difference between *M*_*p*_ and *A*_*p*_. Fig. 4 illustrates that the R-peak and the austenite peak of *NiTi* approach each other with increasing heat treatment temperatures, merging at 530 °C and above. This observation indicates that residual stress in the matrix, which impedes the martensitic transformation, is mitigated during the final heat treatment. The DSC curves reflect this stress relaxation through a steeper and higher M-peak upon cooling, indicating that the stress fields obstructing the martensitic transformation are minimized.

Moreover, high lattice strain in the as-received (cold-worked) condition is known to reduce the energy barrier for R-phase formation [2]. This is consistent with the DSC results, which show that heat treatment at higher temperatures weakens R-phase formation and stabilizes martensitic transformation.

**Fig. 5:**
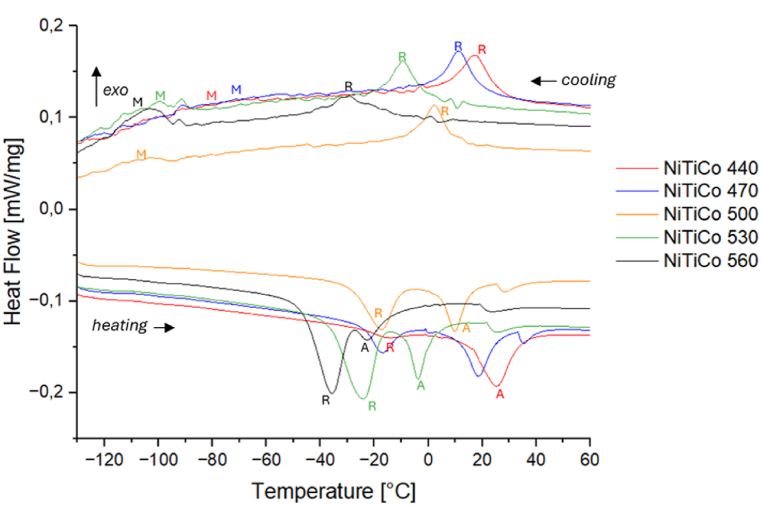
DSC curves of NiTiCo samples heat treated at 470 °C, 500 °C and 530 °C.

The DSC curves of *NiTiCo* alloy follow an analogous trend, as $A_{f}$ shifts towards lower values with increasing heat treatment temperatures (Fig. 5). When the heat treatment temperature is below 500 °C, the martensite peak upon cooling is very broad and the transformation occurs over a wide range of temperatures. At higher heat treatment temperatures, *M*-peak becomes more noticeable, while the *R*-peak becomes smaller.

On the other hand, the heating curves show two distinct peaks which correspond to the *M → R* and *R → A* transformations. A third, small peak is visible around 30 °C in the heating curves. This could be an artifact or could indicate that a residual amount of R-phase is still present in the system at temperatures higher than 20 °C. It may be possible that the austenite transformation occurs in two steps, however, further analysis is needed to understand this.

**Fig. 6:**
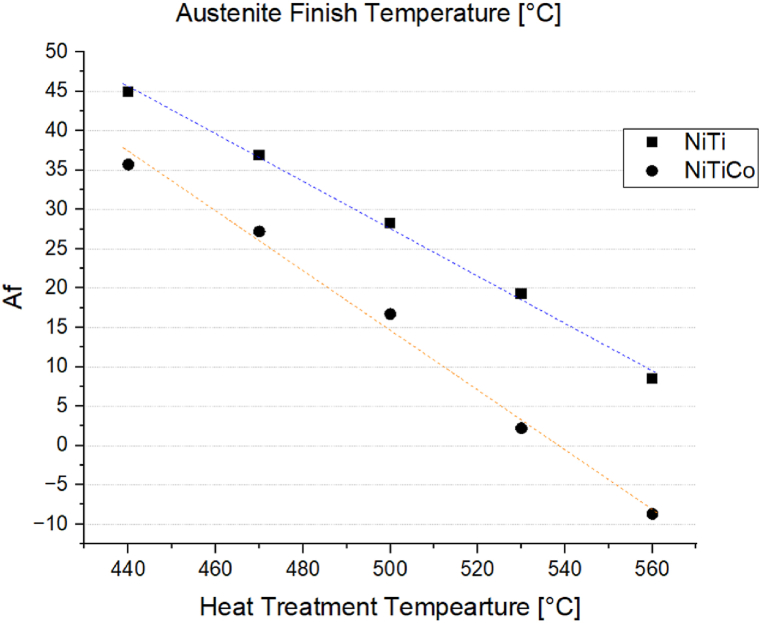
A_f_ after heat treating for 30 min at temperatures between 440 °C and 560 °C.

Fig. 6 shows the austenite phase transition temperature (Af) of *NiTi* and *NiTiCo* specimens heat-treated for 30 min at temperatures ranging from 440 °C to 560 °C. In both the binary (*NiTi*) and ternary (*NiTiCo)* alloys, *Af* decreases with increasing heat treatment temperatures. Additionally, the presence of *Co* accelerates the rate at which *Af* decreases with higher heat treatment temperatures. Pelton et al. [10] suggest that this accelerated decrease in *Af* may be due to the dissolution of dynamic precipitates when heat treating above 500 °C, which results in a reduction of *Af* by increasing the *Ni* content in the matrix. However, EDS analysis does not reveal a clear correlation between the *Ni* content of the matrix and *Af*. Therefore, the observed changes in thermal behaviour could also be attributed to variations in stress fields and concentration inhomogeneities associated with the precipitates.

The results indicate that the performance of the as-received alloy can be finely tuned through careful final heat treatment. For both *NiTi* and *NiTiCo* alloys, the austenite finish temperature (*Af*) decreases significantly with increasing heat treatment temperatures. Specifically, for *NiTi* samples, *Af* drops from 44.9 °C after heat treatment at 440 °C to approximately 8.5 °C after treatment at 560 °C. Conversely, the *Af* for *NiTiCo* specimens ranges from a maximum of 35.7 °C to a minimum of −8.7 °C. The transformation temperatures of *NiTi* and *NiTiCo* specimens heat-treated between 440 °C and 560 °C are summarized in Table 4.

**Table 4:** Transformation temperatures in °C obtained through DSC measurements.

*Material*	$R_{sc}$	$R_{fc}$	$M_{s}$	$M_{p}$	$M_{f}$	$R_{sh}$	$R_{fh}$	$A_{s}$	$A_{p}$	$A_{f}$	*Hysteresis*
*NiTi 440*	36.9	24.1	−28.5	−52.5	−115	4.2	–	–	38.1	44.9	90.6
*NiTi 470*	31.1	21.6	−29.1	−53.2	−117.9	2.8	–	–	27.3	36.9	80.5
*NiTi 500*	21.8	13.5	−26.9	−49.6	−77.6	7.3	–	–	24.3	28.2	73.9
*NiTi 530*	12	0.3	−31	−47	−61	–	–	5.9	13.7	19.3	60.7
*NiTi 560*	0.2	−24.7	−25.7	−47.2	−66.2	–	–	-7–8	0.8	8.5	48
*NiTiCo 440*	29.1	3.5	−32.5	−75.9	−117.2	−28.2	−4.5	14.2	24.9	35.7	100.8
*NiTiCo 470*	20.9	0.8	−38.1	−68.7	−129.4	−26.1	−8.1	9.4	17.9	27.2	86.6
*NiTiCo 500*	10.1	−6.9	−92.8	−106.9	−125.4	−30.1	−9.9	3.5	9.5	16.7	116.4
*NiTiCo 530*	0.8	−19.7	−83.8	−100.4	−121.9	−36.9	−16.6	−9.2	−3.6	2.2	96.8
*NiTiCo 560*	−3.4	−49.3	−91.1	−103.4	−123.8	−48.9	–	–	−23.1	−8.7	80.3

### 3.4 X-ray diffraction (XRD)

**Fig. 7:**
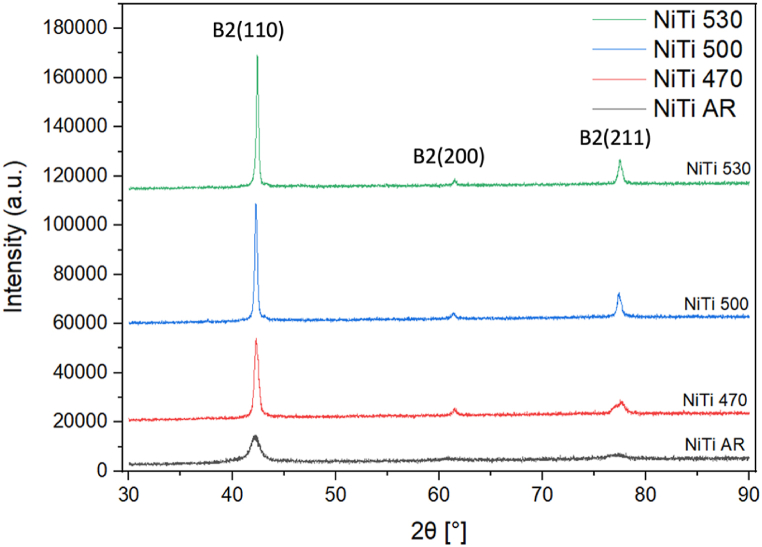
X-ray diffraction patterns of as-received and heat treated NiTi samples.

**Fig. 8:**
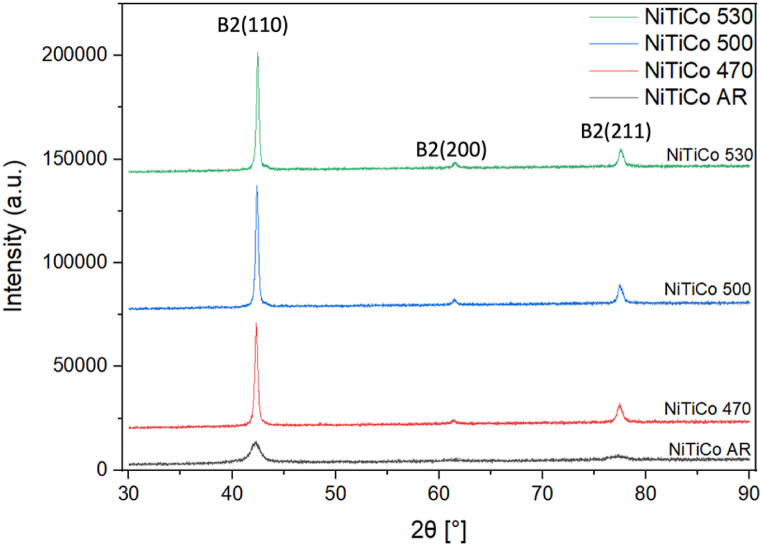
X-ray diffraction patterns of as-received and heat treated NiTiCo samples.

The XRD analysis was conducted isothermally at 20 °C (Fig. 7, Fig. 8) and according to the DSC results the samples are expected to be mainly austenitic. The plot of heat treated *NiTi* samples shows that the peak intensity increases with increasing heat treatment temperature (Fig. 7). All curves have a similar shape, a high and sharp peak around 42.5° which represents the *(110)* plane for austenite [19,26] consistent with the DSC results. For all the XRD patterns of *NiTi* and *NiTiCo* alloys heat treated at 470 °C, 500 °C and 530 °C *(110),* (200) and (211) austenite peaks are visible.

The Williamson-Hall method, which accounts for strain-induced broadening of XRD peaks, was employed to calculate both intrinsic strain and particle size [27,28]. The presence of tensile stress in the material increases the full width at half maximum (FWHM) of XRD peaks, while stress relaxation decreases the FWHM [29]. Additionally, shifts in the austenite peaks were observed.

**Fig. 9:**
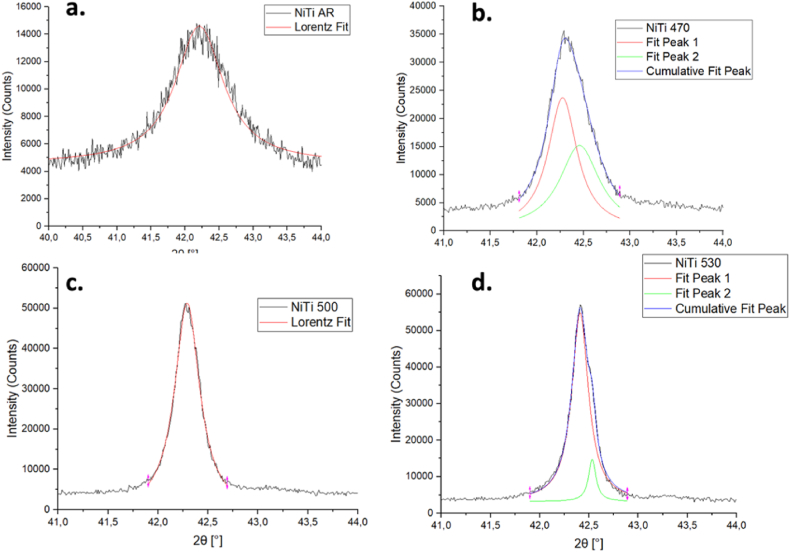
XRD analysis of B2(110) peak in as-received and heat treated NiTi samples.

**Fig. 10:**
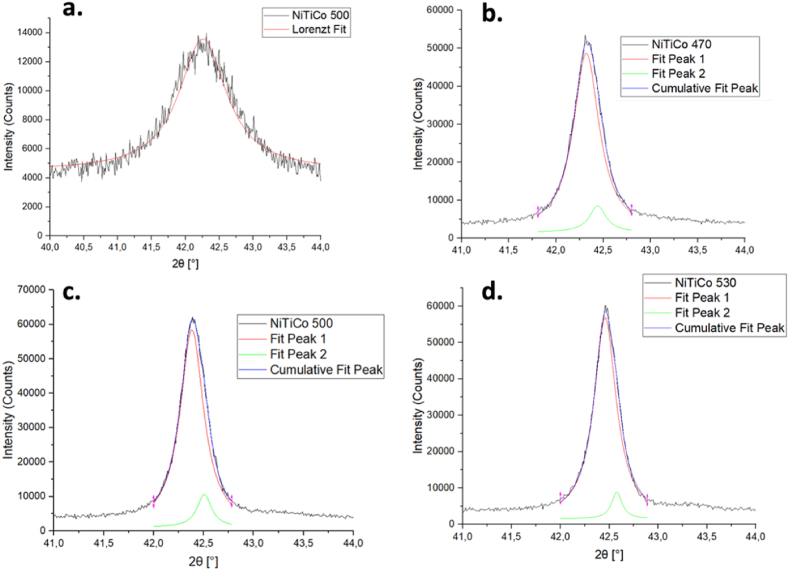
XRD analysis of B2(110) peak in as-received and heat treated NiTiCo samples.

A leftward shift in a peak may indicate lattice expansion, while microstrain in the lattice can alter the d-spacing, impacting the peak position and causing peak broadening (FWHM). Lorentzian peak fitting was performed using OriginPro software to calculate the FWHM, as illustrated in Fig. 9, Fig. 10. In these figures, "Fit Peak 1″ and "Fit Peak 2″ represent the individual Lorentzian fits applied to the experimental data of the B2 (110) peak. The cumulative fit, which is the sum of these two Lorentzian fits, represents the overall fit to the experimental data.

From the detailed analysis of the (110) peak (Fig. 9, Fig. 10), the corresponding values for FWHM and d-spacing are summarized in Table 5. For most specimens subjected to heat treatment, the evolution of secondary peaks was observed. A decrease in FWHM upon heat treatment indicates stress relaxation. The peak positions generally shift to the right with increasing heat treatment temperature, reflecting the reduction in stress. Additionally, d-spacing values were slightly lower for Co-containing alloys at heat treatment temperatures of 470 °C and above. This observation is consistent with the more rapid decrease in Af temperatures observed in the presence of Co in the matrix.

**Table 5:** Tabular documentation of the XRD results.

*Material*	*(110) Peak position*	*d* [Å]	a [nm]	*FWHM*
*NiTi AR*	*42.20*	*2.147*	0.3036	*0.852*
*NiTi 470*	42.27	2.143	0.3031	0.381
*NiTi 500*	42.29	2.142	0.3030	0.307
*NiTi 530*	42.41	2.136	0.3021	0.205
*NiTiCo AR*	42.25	2.144	0.3033	0.848
*NiTiCo 470*	42.32	2.141	0.3028	0.333
*NiTiCo 500*	42.38	2.138	0.3023	0.288
*NiTiCo 530*	42.46	2.134	0.3019	0.276

It is important to address the observed lack of R-phase peaks in the XRD patterns, despite their presence at room temperature as indicated by DSC. Several factors could contribute to this discrepancy. Residual stresses and texture effects can influence XRD peak positions and intensities, potentially obscuring or altering the R-phase peaks. Additionally, the coexistence of austenite and R-phase at room temperature may not be fully resolved by XRD, as the R-phase could be present only in a small fraction or as fine domains that are below the detection limit of the XRD resolution employed. Furthermore, the peak fitting methodology employed for FWHM analysis is more effective for the primary peaks and may not fully capture the subtle characteristics of secondary phases like the R-phase, which could affect the ability to detect and differentiate these phases accurately. In contrast, DSC can detect even small amounts because it is sensitive to changes in heat flow associated with phase transitions rather than the intensity of diffraction peaks.

## 4 Discussion

**Fig. 11:**
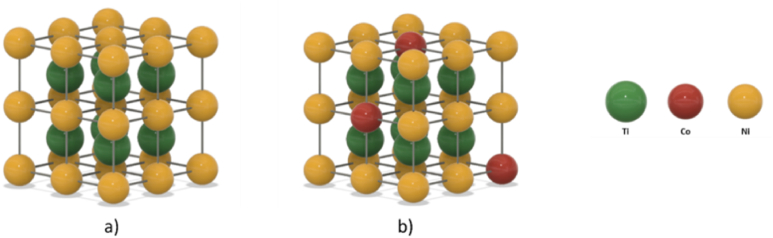
Schematic representation of the B2 structure of NiTi (a) and NiTiCo (b).

The austenite B2 structure in NiTi alloys is an ordered CsCl-type structure [[30], [31], [32]], consisting of two interpenetrating simple cubic sublattices (Fig. 11(a)). The potential effect of *Co* addition is shown in Fig. 11 (b) where it is assumed *Co* atoms preferentially occupy *Ni* sites regardless of alloy composition. By occupying Ni lattice sites and introducing lattice distortions, *Co* addition can affect phase stability and transformation kinetics [18].

The comparison of the atomic radii (Ni:1.25 Å, Co:1.25 Å and Ti:1.45 Å) suggests a decrease in d-spacing upon heat treatment of cold-worked as-received samples can linked with a decrease in Ti-content in the matrix after heat treatment at 470*°*C. If a Ti atom leaves the lattice to form a different phase, such as the Ti-rich precipitates observed in the EDS analysis, the result will be a decrease in distance between the atomic planes. On the other hand, higher heat treatment temperatures such as 530*°*C and above increased the content of Ti in the matrix due to the dissolution of Ti-rich precipitates back into the matrix. However, it was determined that d-spacing values continued to decrease upon heat treatment at 500*°*C and above. Therefore, the continuous *d*-spacing decrease by the increase in heat treatment temperature cannot be directly linked with an increase in Ti content in the dissolved state at 530*°*C. It can be stated that structural relaxation is the main controlling factor for lattice shrinkage where the effects of compositional variation are not clear.

**Fig. 12:**
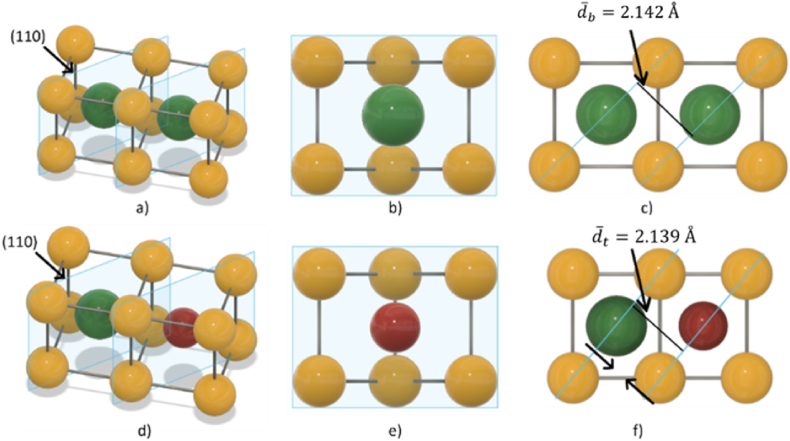
Schematic representation of (110) planes, cross section along the plane (110), and the top view of NiTi (a, b, c) and NiTiCo (d, e, f) structures. The top view allows to clearly see the different d-spacing between the two alloys. The addition of Co changes the solubility of Ti in the system, reducing the spacing between the (110) planes, as confirmed by XRD analysis.

In this study, it is suggested that the addition of *Co* results in faster structural relaxation leading to a more pronounced decrease in Af temperatures than binary alloy. XRD analysis supports this finding that *d*-spacing values of *NiTiCo* samples heat treated at 500 °C and above are slightly lower than the binary alloy. When *Co* is added, Ti solubility decreases up to 530*°*C leading to a greater number of Ti atoms being extracted from their lattice sites leaving behind vacancies which could be filled by Co atoms. Fig. 12 shows a potential mechanism of lattice change upon *Co* addition. However, at 530 °C, even with the increase in Ti-content, d-spacing values continue to decrease, hence, lattice shrinkage is controlled by structural relaxation independent of compositional change, i.e. Ti-content increase.

The addition of *Co* affects the precipitation kinetics both during the thermomechanical processing and the final heat treatment. It is determined that larger and fewer precipitates were present in *NiTiCo* samples compared to the ones observed in the NiTi specimens. Independent of the heat treatment temperature and the alloy, all the particles observed are Ti-rich precipitates, which were already present in the as-received samples. Hence, the heat treatment temperature does not affect the nature of the observed phases in this case. With increasing heat treatment temperature at 500 °C and above, the size of the particles decreases in both *NiTi* and *NiTiCo* alloys. This is probably due to the higher solubility of Ti in the system at higher temperature leading to the dissolution of the Ti-rich particles.

XRD analysis further indicates that the heat treatment temperature affects FWHM, which is associated mainly with the micro-strain in the lattice of the material under investigation. According to the Williamson-Hall method, the relaxation of tensile stress decreases the FWHM. Since the peak width decreases with increasing heat treatment temperatures for both *NiTi* and *NiTiCo* samples, the results suggest that stress fields in the system are mostly relieved through heat treatment. More importantly, lower FWHM were determined for Co containing alloys at temperatures 500*°*C and above except the highest temperature (530*°*C) indicating less internal stresses, and faster stress relaxation compared to nitinol consistent with DSC analyses. At 530*°*C, peak-splitting occurred which increased FWHM calculation.

The DSC analyses show that in both binary and ternary alloys, $A_{f}$ temperature decreases significantly with increasing heat treatment temperature. Even though this was reported previously, the rate of change is determined to be higher for *Co* containing system. Heat treatment at 440 °C results in $A_{f}$ of 44.9 °C and 35.7 °C for *NiTi* and *NiTiCo* samples, respectively. On the other hand, *NiTi* and *NiTiCo* complete their transition to austenite at 8.5 °C and −8.7 °C, respectively when heat treated at 560 °C. However, the analysis of the matrix composition indicates that this change in $A_{f}$ with increasing heat treatment temperatures is independent of Ni/Ti ratio or compositional variation. Hence, the change in the thermal behaviour of the material is determined to be mainly due to the stress relaxation which may be associated with a decrease in dislocation density.

## 5 Conclusion

In this study, the effects of Co addition and different heat treatment temperatures on the transformation behaviour of NiTi alloys were investigated. The results are summarized as follows.

• The examination of the SEM micrographs shows that *Co* affects the precipitation kinetics, resulting in the presence of larger particles compared to the binary alloy. It is suggested that *Co* reduces the solubility of *Ti* in the system which leads to the extraction of *Ti* from the matrix and the formation of larger *Ti*-rich particles.
• Given that only Ti-rich phases can be observed in the samples, it is likely that these precipitates form during the manufacturing process. An analysis of the exact processing steps which induce the precipitation of such phases needs to be carried out to have a better control of particle formation.
• DSC analysis shows that the $A_{f}$ temperature decreases with increasing heat treatment temperature for both *NiTi* and *NiTiCo* specimens where the decrease rate is higher for *Co* containing system.
• The DSC curves of the *NiTiCo* samples reveal the presence of a three-peak curve upon heating. At this stage, it is unclear whether the third peak is an artifact, or it is due to the presence of a residual amount of *R-*phase which only completes the transformation at temperatures close to 36 °C.
• When Co is present, faster structural relaxation with increasing heat treatment temperature is found and evidenced by the variation in lattice size.

Since the addition of Co changes the transformation temperatures of Ni-Ti based alloy system, the heat treatment methodology needs to be tailored to the specific alloy composition for controlling superelasticity and SME via alloy design.

## CRediT authorship contribution statement

**Rocco Puopolo:** Writing – review & editing, Writing – original draft, Methodology, Investigation, Formal analysis, Data curation, Conceptualization. **Sally Ruschendorf:** Writing – original draft, Methodology, Investigation, Formal analysis, Data curation, Conceptualization. **Ajai S.K. Thadayil:** Writing – review & editing, Methodology, Formal analysis, Data curation. **Scott Cook:** Writing – review & editing, Supervision, Project administration. **Mert Celikin:** Writing – review & editing, Supervision, Methodology, Investigation, Conceptualization.

## Declaration of competing interest

The authors declare that they have no known competing financial interests or personal relationships that could have appeared to influence the work reported in this paper.
