# Supplementary material for: The effect of cobalt alloying on the phase transformation kinetics of Ni-Ti alloys
Source: Heliyon. 2024 Sep 3;10(18):e37390. doi: 10.1016/j.heliyon.2024.e37390 (PMC11416287; doi:10.1016/j.heliyon.2024.e37390)

Below are details about the image analysis procedure on a representative sample (NiTiCo 470):


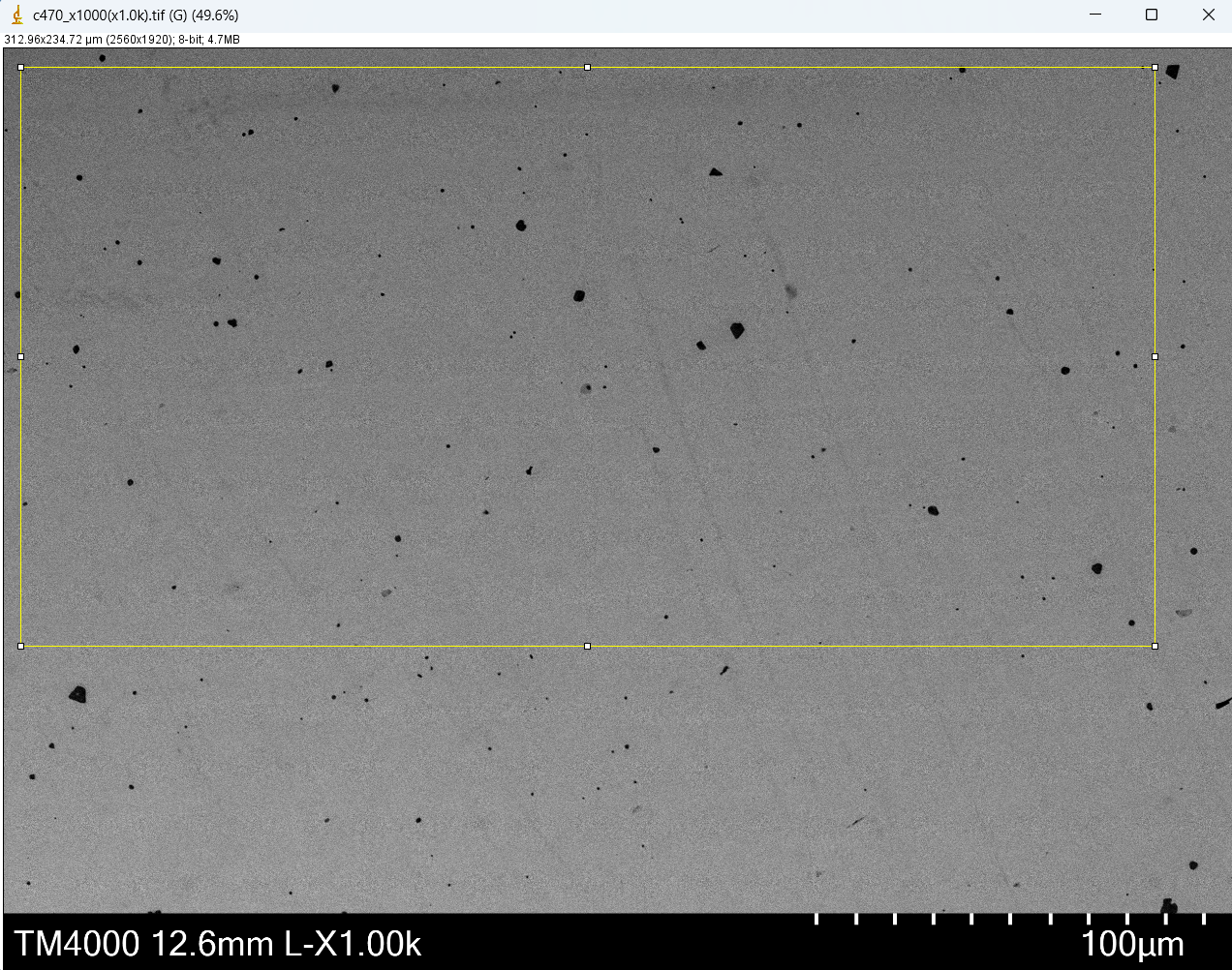

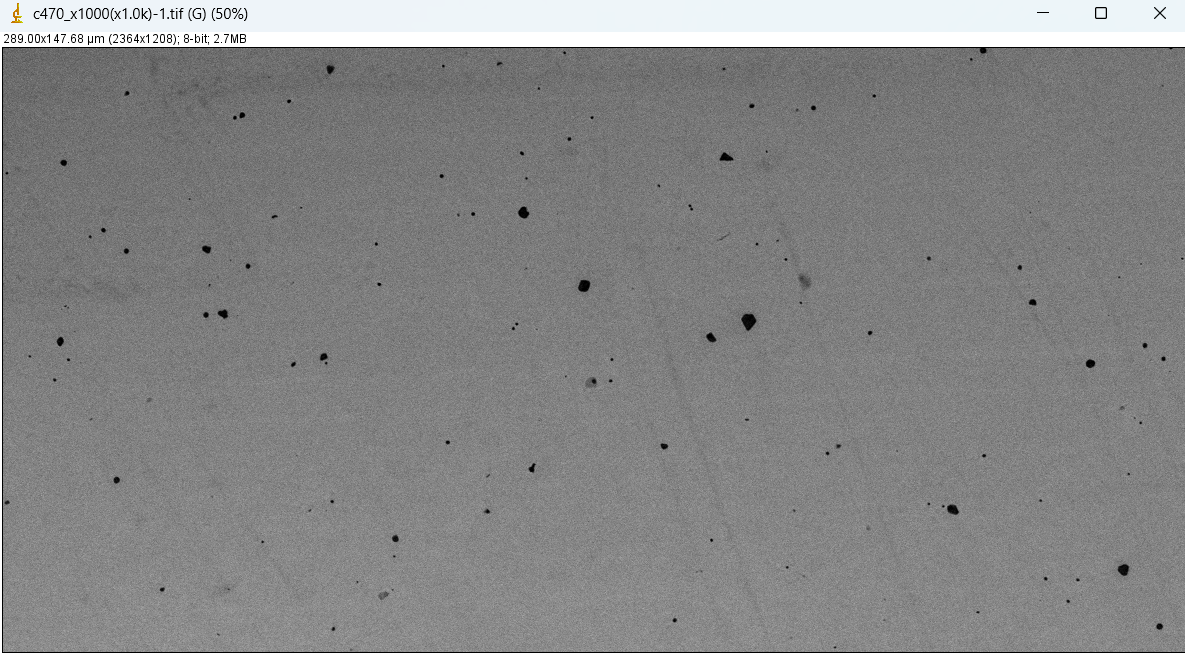


Process 🡪 FFT 🡪 Bandpass filter


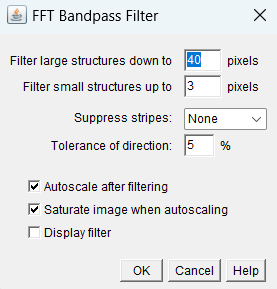

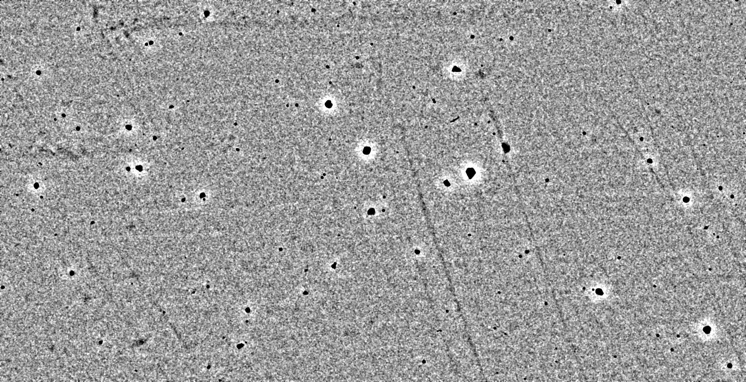


Image 🡪 Adjust 🡪 Threshold


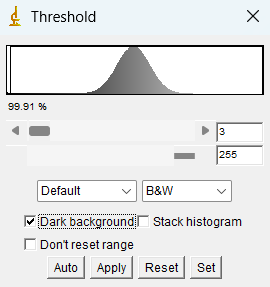

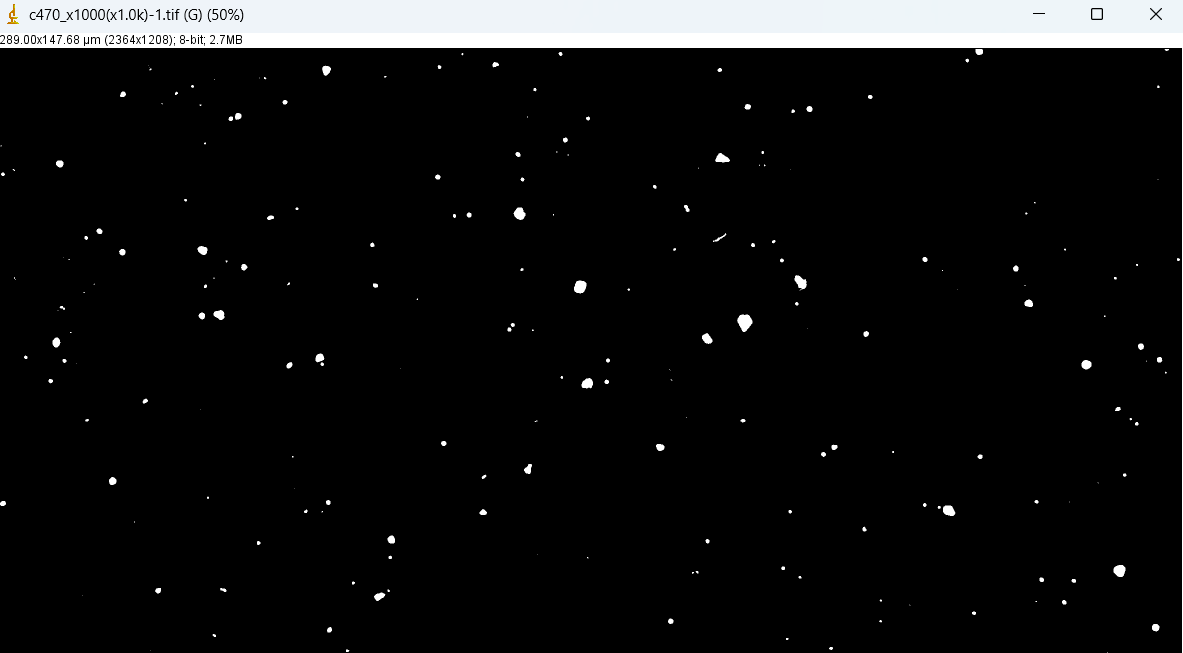


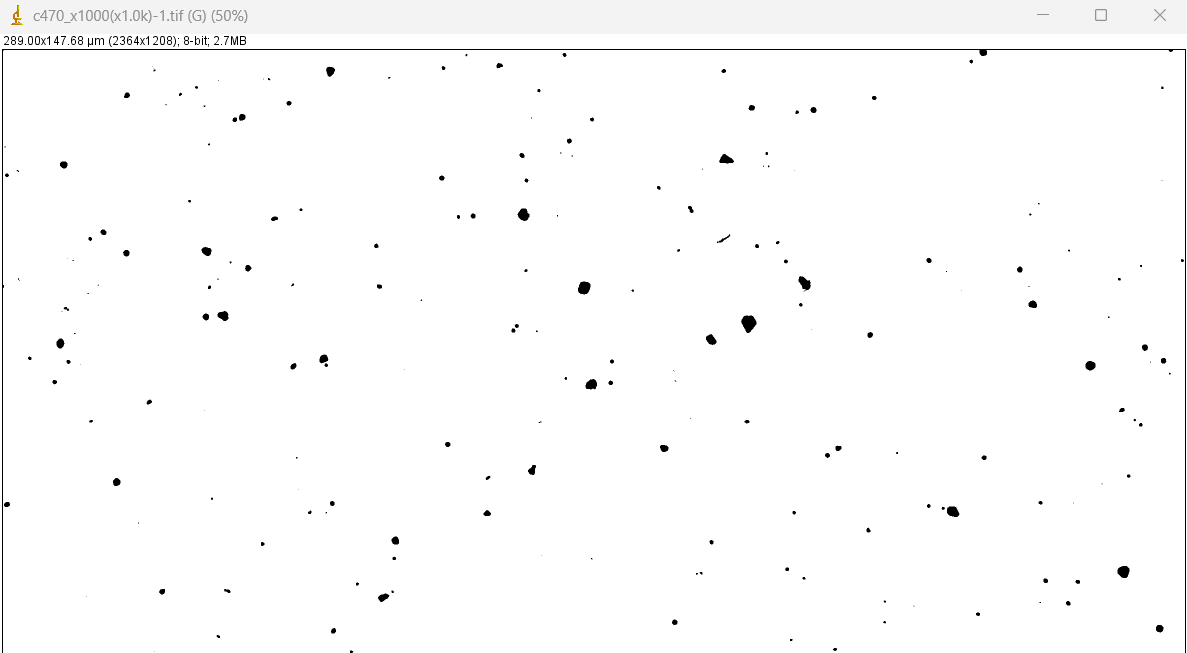


|  | **Number of Particles** | **Average Size** | **Standard Deviation** | **Variance** |
| --- | --- | --- | --- | --- |
| NiTiCo 470 | 199 | 1,15 μm | 0.8 μm | 0.64 μm |

**Average Sizes and number of particles used per condition**

|  | **Number of Particles** | **Average Size [μm]** | **Standard Deviation (σ)** | **Variance**  **(σ^2^)** |
| --- | --- | --- | --- | --- |
| **NiTi AR** | 376 | 0.55 | 0.22 | 0.05 |
| **NiTi 470** | 300 | 0.68 | 0.24 | 0.06 |
| **NiTi 500** | 409 | 0.57 | 0.26 | 0.07 |
| **NiTi 530** | 375 | 0.30 | 0.22 | 0.05 |
| **NiTiCo AR** | 324 | 0.84 | 0.50 | 0.25 |
| **NiTiCo 470** | 254 | 1.1 | 0.51 | 0.26 |
| **NiTiCo 500** | 398 | 0.93 | 0.47 | 0.22 |
| **NiTiCo 530** | 383 | 0.77 | 0.40 | 0.16 |

Detailed Peak Fitting of B2 (110) with functions


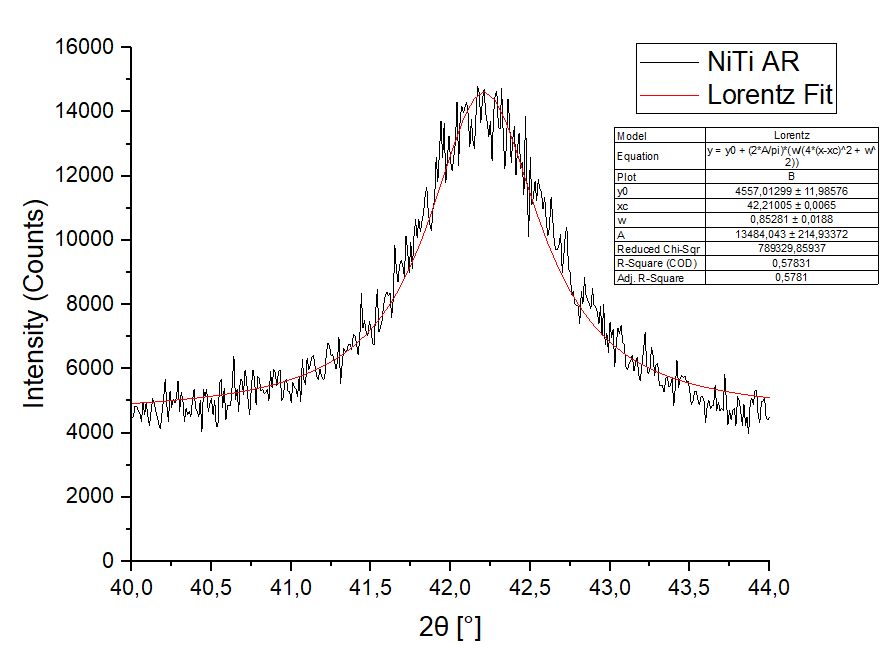


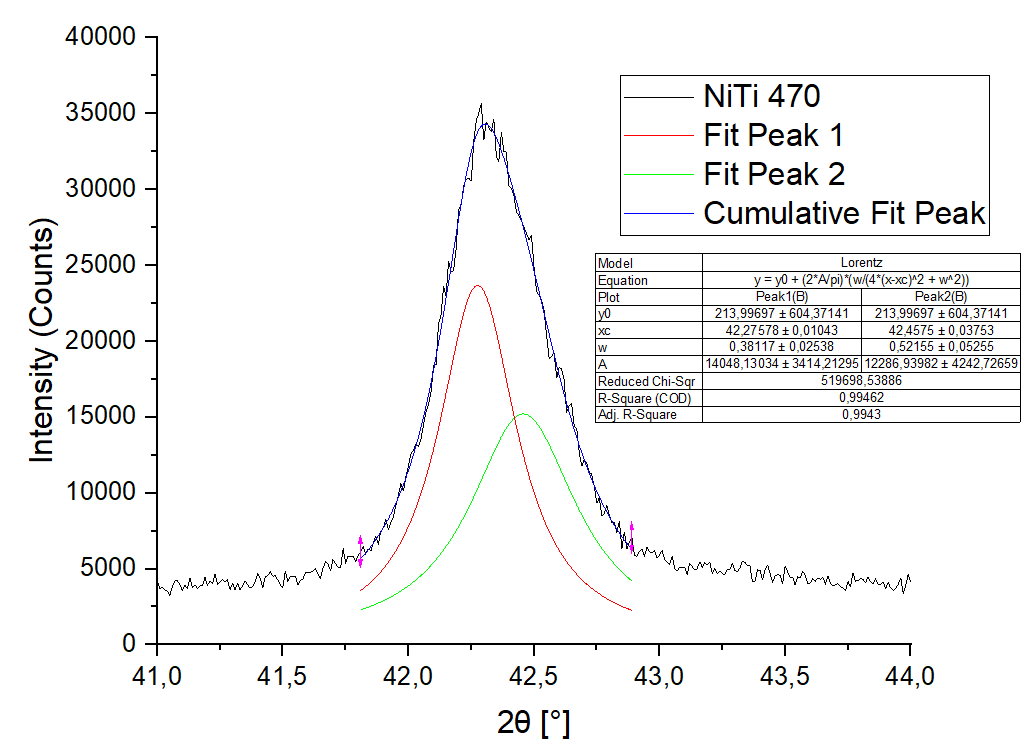


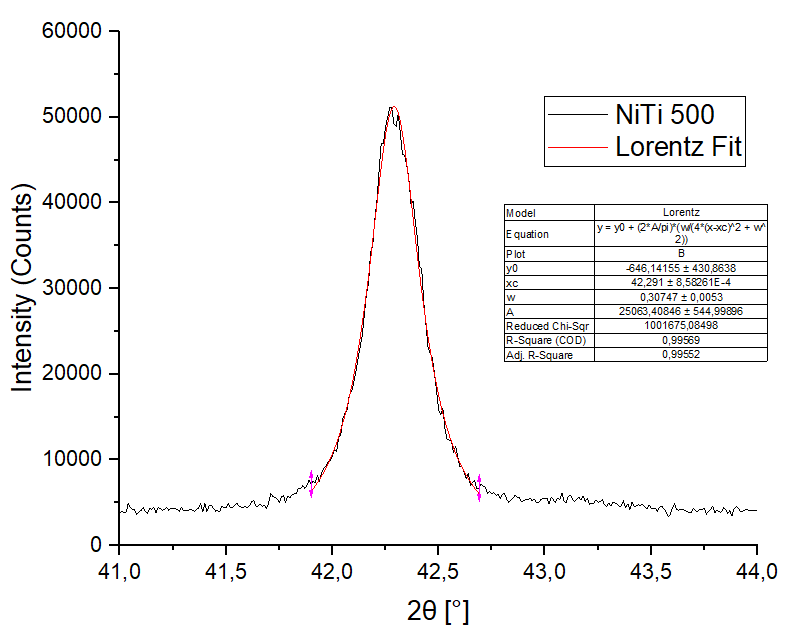


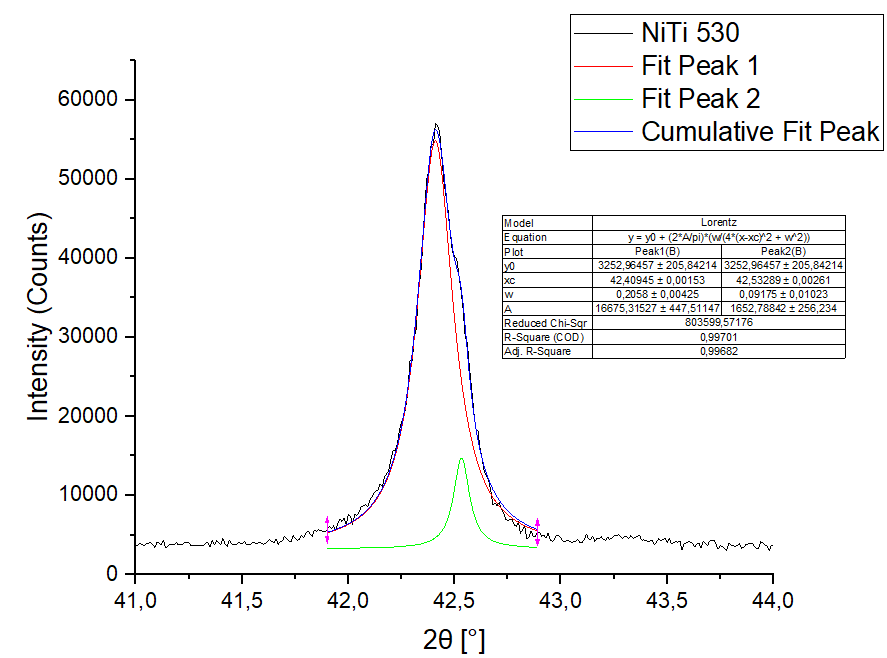


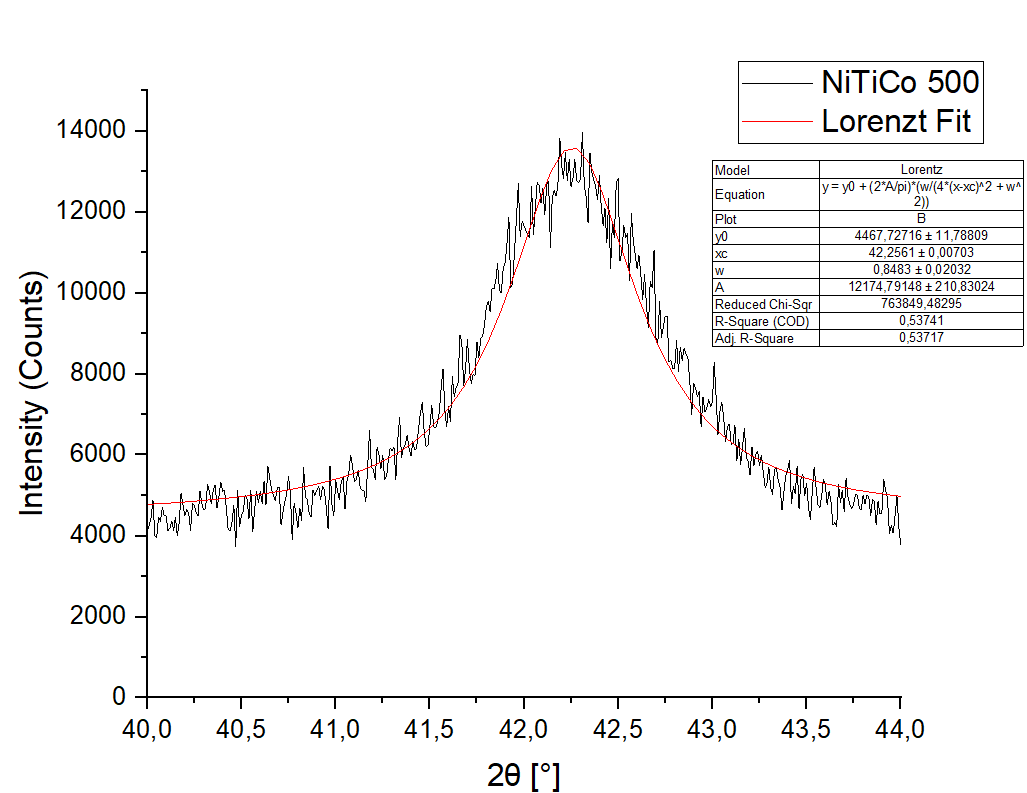


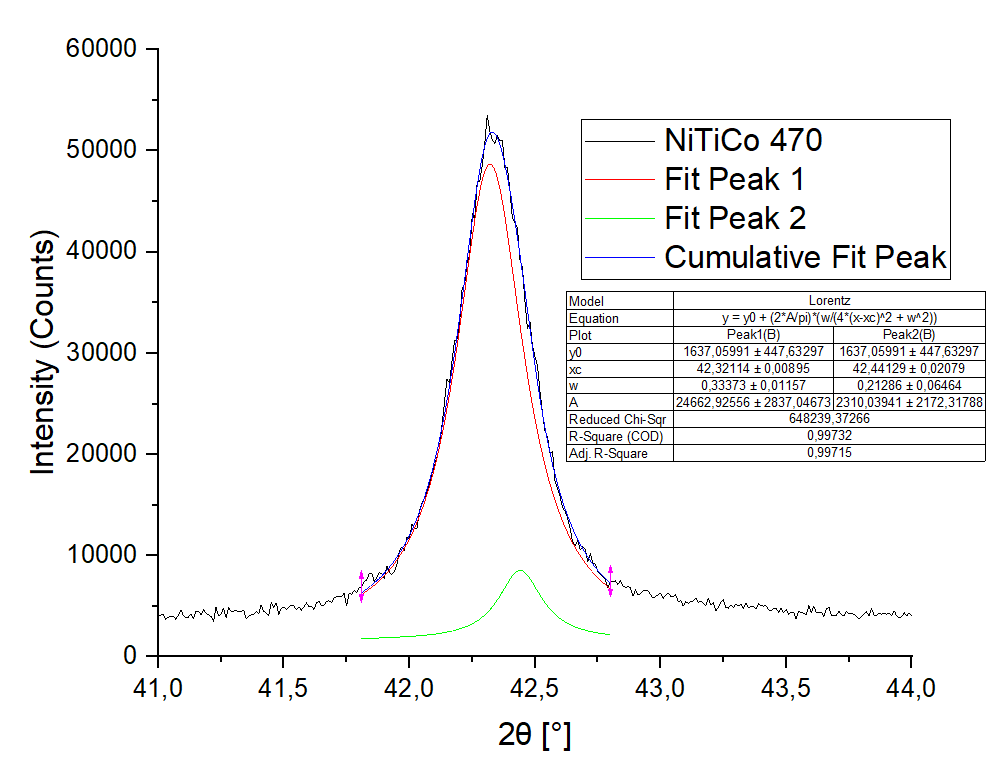


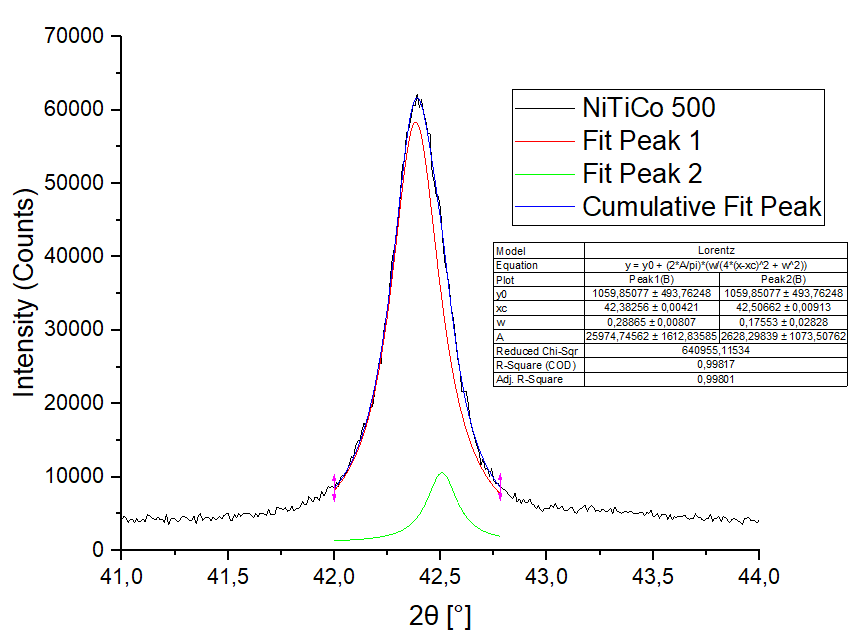


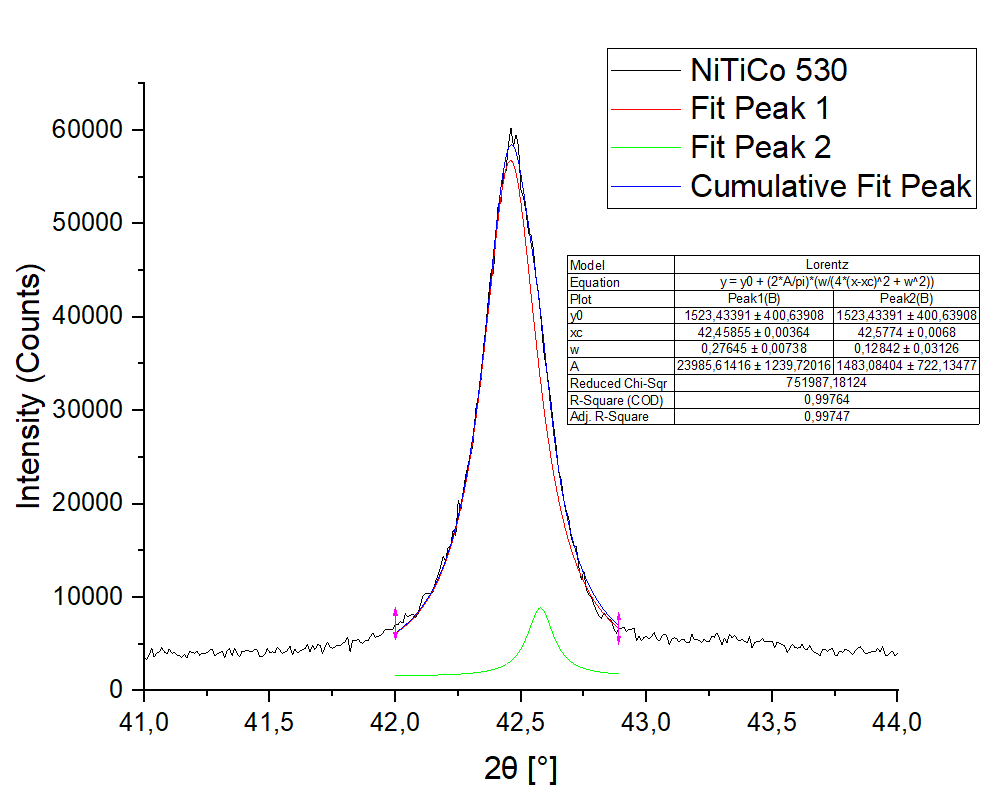

Supplement: Multimedia component 1 [file mmc1.docx]
